# Postactivation Potentiation of the Plantar Flexors Does Not Directly Translate to Jump Performance in Female Elite Young Soccer Players

**DOI:** 10.3389/fphys.2018.00276

**Published:** 2018-03-23

**Authors:** Olaf Prieske, Nicola A. Maffiuletti, Urs Granacher

**Affiliations:** ^1^Division of Training and Movement Sciences, Research Focus Cognition Sciences, University of Potsdam, Potsdam, Germany; ^2^Human Performance Lab, Schulthess Clinic, Zurich, Switzerland

**Keywords:** sensorimotor training, conditioning activity, twitch torque, power, soccer

## Abstract

High-intensity muscle actions have the potential to temporarily improve muscle contractile properties (i.e., postactivation potentiation, PAP) thereby inducing acute performance enhancements. There is evidence that balance training can improve performance during strength exercises. Taking these findings together, the purpose of this study was to examine the acute effects of a combined balance and strength (B+S) exercise vs. a strength only (S) exercise on twitch contractile properties, maximum voluntary strength, and jump performance in young athletes. Female elite young soccer players (*N* = 12) aged 14–15 years conducted three experimental conditions in randomized order: S included 3 sets of 8–10 dynamic leg extensions at 80% of the 1-repetition maximum, B+S consisted of 3 sets of 40 s double-leg stances on a balance board prior to leg extensions (same as S), and a resting control period. Before and 7 min after exercise, participants were tested for their electrically-evoked isometric twitches (i.e., twitch peak torque, twitch rate of torque development) and maximal voluntary contraction (MVC) torque of the plantar flexor muscles. Additionally, countermovement (CMJ) and drop jump (DJ) performances (i.e., CMJ/DJ height, DJ ground contact time) were assessed. Significant effects of condition on twitch contractile properties (*p* < 0.05, *d* = 1.1) and jump performance outputs (*p* < 0.05, 1.1 ≤ *d* ≤ 1.2) were found. *Post-hoc* tests revealed that S compared to control produced larger PAP for twitch peak torques by trend (*p* = 0.07, *d* = 1.8, 33 vs. 21%) and significantly larger PAP for twitch rate of torque development (*p* < 0.05, *d* = 2.4, 55 vs. 43%). Following B+S compared to control, significant improvements in CMJ height (*p* < 0.01, *d* = 1.9, 3%) and DJ contact time were found (*p* < 0.01, *d* = 2.0, 10%). This study revealed protocol-specific acute performance improvements. While S resulted in significant increases in twitch contractile properties, B+S produced significant enhancements in jump performance. It is concluded that PAP effects in the plantar flexors may not directly translate to improved jump performance in female elite young soccer players. Therefore, the observed gains in jump performance following B+S are most likely related to neuromuscular changes (e.g., intramuscular coordination) rather than improved contractile properties.

## Introduction

Short bouts of high-intensity exercise induce fatigue and have the potential to acutely improve the muscle's capability of generating high forces over a short period of time, which is usually denoted in the literature as postactivation potentiation (PAP) (Sale, 2002). Alterations in subsequent strength and power output may represent net performance changes due to fatigue and/or potentiation (Tillin and Bishop, 2009). PAP effects may also translate into performance enhancements of physical fitness components (Sale, 2002; Tillin and Bishop, 2009). For instance, Kilduff et al. (2008) reported that countermovement jump height was significantly enhanced by 5% following a loaded squat exercise [i.e., 3 sets of 3 repetitions at 3-repetition maximum (RM)] in male elite rugby players aged 25 years. In another study, Low et al. (2015) observed that repeated sprint performance significantly improved (~1%) following loaded back squats (91% of 1-RM) in male adolescent soccer players aged 17 years. A recent meta-analysis summarized the acute effects of different types of exercise on subsequent power output in athletes (Wilson et al., 2013). More specifically, Wilson et al. (2013) reported large effect sizes for power enhancement following squat and leg press exercises in athletes (standardized mean difference = 0.81). Consequently, strength exercise (e.g., leg press with 80% 1-RM) appears to be an appropriate means to induce short-term performance enhancements (e.g., jump height) in male (young) athletes. However, there is no study available that has examined the effects of strength exercises on subsequent jump performance in female young soccer players.

Further, it has been reported that balance exercises have the potential to modulate neural activation of the plantar flexor muscles. In fact, cross-sectional studies revealed lower H-reflex amplitudes during the performance of complex postural tasks (e.g., walking on unstable balance beams) compared with tasks that afforded low complexity (e.g., walking on a treadmill) in healthy young adults (Llewellyn et al., 1990; Trimble et al., 2000; Chalmers and Knutzen, 2002; Day et al., 2017). This indicates diminished spinal excitability of the monosynaptic reflex pathway. Moreover, Horslen et al. (2013) found higher Achilles tendon stretch reflex responses under unstable compared to stable conditions (i.e., standing on a tilting platform vs. a firm surface). It was suggested that the observed increases in afferent feedback are related to better muscle spindle sensitivity. Taube et al. (2007) translated these findings to an intervention study. H-reflex amplitudes were significantly reduced after 6 weeks of balance training in adolescent athletes with a mean age of 15 years. In fact, spinal and cortical excitability are reduced following balance training, whereas activity in subcortical regions may increase (Taube et al., 2008). These changes can contribute to more coordinated and/or automatized muscle activation during motor performance (e.g., leg extensions) (Taube et al., 2008). In fact, Taube et al. (2007) observed a balance training induced trend for increased leg muscle activity (e.g., gastrocnemius muscle) in the range of 29–54% during maximal isometric leg extensions. Moreover, following 4 weeks of balance training in healthy young adults, Gruber et al. (2007) reported significant increases in electromyographic median frequency of the triceps surae muscle during maximal isometric plantar flexions (13–45%). These findings from cross-sectional and training studies can possibly be translated to research examining the effects of different types of exercise on acute performance enhancement. In other words, performing balance exercises prior to strength exercises may enhance the potential to improve muscle contractile properties and performance due to a more coordinated muscle activation (e.g., inter-/intramuscular activation) during strength exercises (e.g., leg extensions). To our knowledge, however, no study has previously investigated whether balance exercise performed prior to strength exercise has the potential to acutely facilitate twitch contractile properties (i.e., PAP) and/or jump performance compared to strength only exercise in female young athletes.

Thus, the purpose of the present study was to examine the acute effects of combined balance and strength (B+S) vs. strength only (S) exercise on twitch contractile properties of the plantar flexor muscles, jump performance, and maximum voluntary strength in female elite young soccer players. With reference to the relevant literature (Gruber et al., 2007; Taube et al., 2007; Wilson et al., 2013), we hypothesized that PAP effects and performance enhancements would be more pronounced following B+S compared to S.

## Methods

### Participants

Twelve healthy female elite young soccer players aged 14–15 years (body height: 166.3 ± 4.3 cm; body mass: 55.1 ± 5.5 kg; body fat: 17.9 ± 5.6%) volunteered to participate in this study. All study participants were members of a soccer team that completed the season with the German under-17 championship title. With reference to a meta-analysis on the acute effects of strength exercises on proxies of muscle power (Wilson et al., 2013), an a priori power analysis with a type I error rate of 0.05 and 80% statistical power was computed. The analysis indicated that 12 young soccer players are sufficient to observe a medium-sized main effect (Cohen's *d* = 0.5) of condition with multiple sets of high-intensity exercise and rest intervals of ~7 min on the primary outcome jump performance. Participants' maturity status was determined by calculating years from peak height velocity (PHV) according to the sex-specific formula that was introduced by Mirwald et al. (2002). Ten soccer players were classified as around-PHV (−1 to +1 years from PHV), while two players were defined as post-PHV (>1 to +3 years from PHV) (Hammami et al., 2016). In addition to physical education classes, participants were engaged in supervised competitive soccer training on a regular basis (including unspecific training for muscular endurance or flexibility) with a mean weekly training volume of 9–10 h. None of the participants suffered from acute musculoskeletal, neurological, or orthopedic disorders that might have affected their ability to execute the experimental protocol. The study was approved by the ethics committee of the University of Potsdam (application no. 28/2015). Prior to the start of the study, written informed consent was obtained from all study participants and their legal representatives. All experiments were conducted according to the latest version of the declaration of Helsinki.

### Experimental procedure

**Figure 1:**
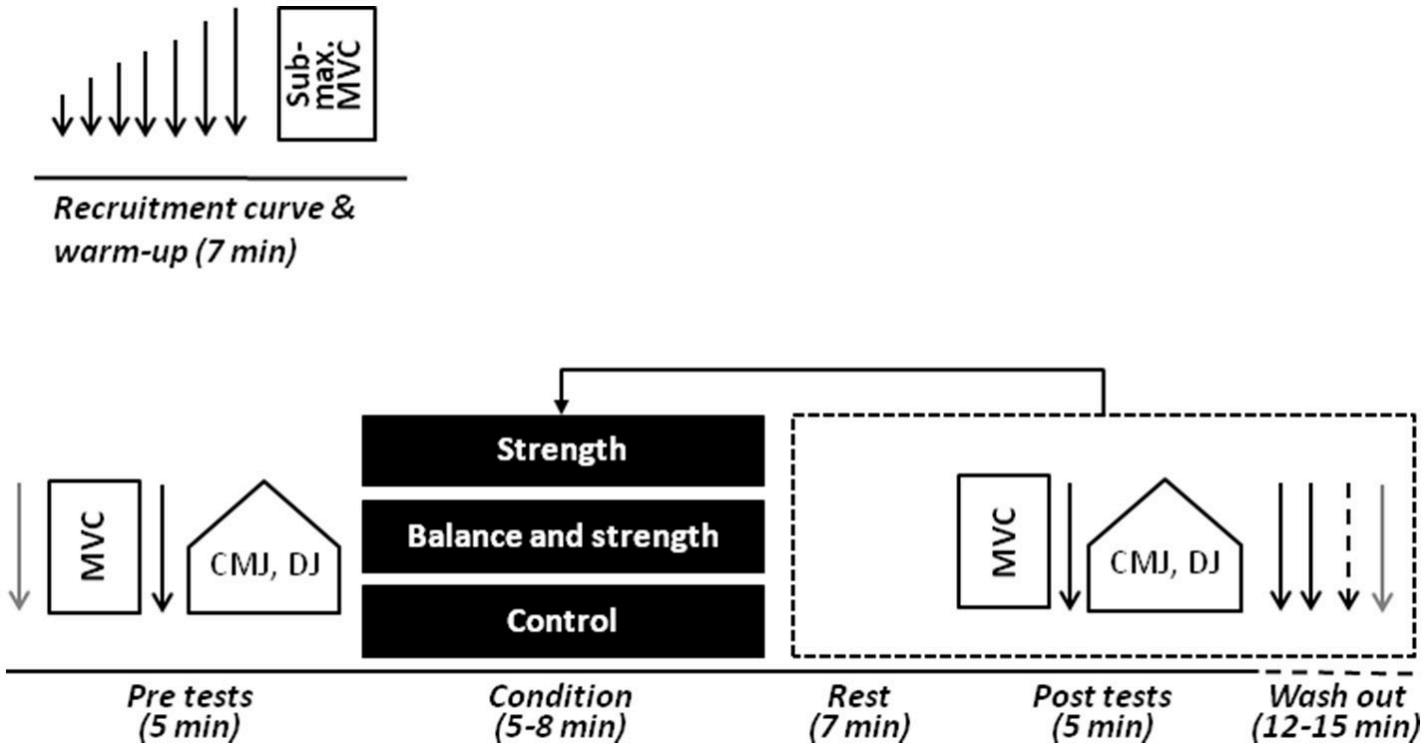
Experimental protocol. The order of experimental conditions (i.e., exercise) was randomized within the same session. Following exercise, a passive rest, post-tests, and a wash-out phase were provided. Arrows depict the application of electrical muscle stimulation (gray arrows = resting twitches). CMJ, countermovement jumps; DJ, drop jumps; MVC, maximal voluntary contraction.

A single group, randomized cross-over design was used to examine the acute effects of exercise on twitch contractile properties and maximal voluntary strength of the plantar flexor muscles as well as jump performance (Figure 1). Measurements were conducted on a single session at the beginning of the season (i.e., pre-season). To get accustomed to the experimental procedures (e.g., dynamometry), one familiarization session was conducted on a separate occasion before the start of the study. During the familiarization session, participants' body height was assessed using a wall-mounted scale. In addition, body mass and percent body fat were quantified by means of a bioelectrical impedance analysis system (InBody 720, BioSpace, Seoul, Korea). Further, an isokinetic dynamometer (Isomed 2000, D&R Ferstl GmbH, Hemau, Germany) was individually adjusted with the participants lying in the supine position with hip, knee, and ankle joints in neutral position (180°, 180°, and 90°, respectively). Previously, Gago et al. (2017) recommended to use an extended knee position (i.e., 180°) for more pronounced PAP effects. The foot of the dominant leg was firmly attached to the lever arm of the dynamometer with its rotational axis at the level of the malleoli. Foot dominance was assessed using the lateral preference inventory (Coren, 1993). In order to limit upper body contribution to torque production, straps/pads were applied at the hip and shoulder level. This fixed position on the dynamometer was maintained throughout the entire test procedures (i.e., for twitch contractile properties and maximal voluntary strength). Additionally, the individual level of instability was determined on an adjustable balance board system (ARTZT vitality Wobblesmart, ARTZT, Dornburg, Deutschland). The balance board (i.e., balance cone) was unstable in 2 dimensions (i.e., frontal and sagittal plane). The base of support was progressively reduced (six levels available) and thus task difficulty increased by means of a pivot that moved outward of the frame producing a convex base of support. The individual level of task complexity/instability was defined as the smallest base of support that an individual was able to successfully accomplish during bipedal stance for 20 s (i.e., hands akimbo, no frame-ground contact). Finally, the leg press 1-RM (126 ± 19 kg, range: 104–158 kg) was determined for each participant as a reference value for the subsequent exercises (Baechle and Earle, 2008).

The experimental session started by searching the maximal stimulus intensity for electrical muscle stimulation, i.e., the intensity where a plateau in peak twitch torque was reached in a relaxed condition. Twitch torque recruitment curves relating evoked torque to stimulation intensity were obtained by delivering single electrical stimuli of increasing intensity (Neyroud et al., 2015). Subsequently, a brief warm-up was completed just before the pre-tests for twitch contractile properties, maximal voluntary strength and jump performance. The warm-up consisted of 8–10 submaximal isometric plantar flexions at 20–80% of maximum voluntary contraction (MVC) torque (~5 min) (Neyroud et al., 2015). Following the pre-tests, three different experimental conditions (S, B+S, control) were organized in a randomized order. Each condition was followed by post-tests for twitch contractile properties, maximal voluntary strength and jump performance (same procedure as pre-tests). Seven minutes of rest were provided upon completion of the condition and the respective post-test, so as to obtain the most efficient net effect of fatigue and potentiation on the assessed strength and jump variables (Lesinski et al., 2013). A wash-out phase (~12–15 min) was provided after each set of the post-tests until twitch torque values returned to baseline (Figure 1).

### Experimental conditions

The three experimental conditions comprised S exercise, B+S exercise, and a passive control condition. S consisted of bilateral eccentric-concentric contractions on a horizontal leg press machine (Eagle leg press, Cybex Int., Medway MA, USA). Participants performed three sets of 8–10 repetitions with a load corresponding to 80% of the 1-RM (i.e., 101 ± 15 kg, range: 83–126 kg), and rest periods of 1–2 min between sets. According to Baechle and Earle (2008), 80% of the 1-RM generally corresponds to the 8-RM load. However, repetitions were not performed until failure. The range of motion for knee and ankle joints amounted to 90–175° and 80–100°, respectively. Time under tension during each repetition (1.5 s eccentric phase, 1.5 s concentric phase) was controlled using an electronic metronome. This training protocol proved to be effective in eliciting PAP effects (Lesinski et al., 2013). During B+S, participants performed three sets of double-leg stance balance exercise (eyes open, hands akimbo) on the adjustable balance board for 40 s, with a rest of 20 s between sets (Lesinski et al., 2015). The level of instability was adjusted according to the individual proficiency on the balance board. After balance exercise, leg press exercise was performed similarly to the S condition. During the passive control condition, participants were asked to rest in a seated position for 8 min. This time interval corresponded to the time needed to complete the B+S condition.

### Assessment of twitch contractile properties

Twitch contractile properties of plantar flexor muscles were determined by means of electrical muscle stimulation and dynamometry. Excellent test-retest reliability for measures of lower limb twitch contractile properties was reported with an intraclass correlation coefficient (ICC) ranging from 0.85 to 0.93 (Place et al., 2007). Single stimuli were delivered transcutaneously to the plantar flexor muscles of the dominant leg via two 5 × 10 cm rectangular self-adhesive surface electrodes (Compex®, DJO France/Division Compex Sport, Mouguerre, France). The anode was placed over the gastrocnemius muscle (~5 cm distal to the popliteal fossa) and the cathode over the soleus muscle (~10 cm proximal to the calcaneus) (Neyroud et al., 2015). Rectangular-wave pulses (200 μs duration) were generated by a high-voltage (max 400 V) constant-current stimulator (Digitimer DS7AH, Hertfordshire, UK). Stimulus intensity was set at 120% of maximal intensity (171 ± 18 mA, range 144–198 mA). As described above, the dynamometer was individually adjusted with the participants lying in the supine position with hip, knee, and ankle joints in neutral position (180°, 180° and 90°, respectively). During pre- and post-tests, twitches were evoked at rest before each experimental condition (i.e., resting twitch) and 2 s after each MVC (i.e., potentiated twitch). The twitch torque signal of the dynamometer was analog-to-digital converted (TeleMyo 2400R G2 Analog Output Receiver, Noraxon®, Scottsdale, AZ, USA), sampled at 1,500 Hz, and stored on a computer running MyoResearch XP Master Edition software (version 1.08.17, Noraxon®, Scottsdale, AZ, USA). Twitch peak torque (twitch PT) and twitch rate of torque development (twitch RTD) were determined from the twitch torque-time curve as the highest torque and the maximal slope between onset of torque and PT, respectively (mean of three trials).

### Assessment of maximal voluntary strength

MVC of the plantar flexor muscles of the dominant leg were conducted on the isokinetic dynamometer as described above. Previously, excellent test-retest reliability has been shown for voluntary MVC torque and RTD in leg extensors (0.80 ≤ ICC ≤ 0.96) (Jenkins et al., 2014). Participants performed 3 MVCs each lasting 3 s while they were consistently encouraged to contract “as forcefully and as fast as possible.” Trials with an identified initial countermovement were discarded after visual inspection of the torque-time curve. The torque signal was analog-to-digital converted, sampled at 1,500 Hz, and analyzed using MyoResearch XP Master Edition software. MVC torque and voluntary RTD were defined as the highest torque and maximal slope between onset of torque and PT of the torque-time curve, respectively (mean of three trials). For each experimental condition, post-test values were expressed as a percentage relative to pre-test.

### Assessment of jump performance

To assess jump performance, participants performed maximal vertical countermovement jumps (CMJ) and drop jumps (DJ) on a three-dimensional force plate (type 9286AA; Kistler®, Winterthur, Switzerland). Excellent test-retest reliability was previously reported for the CMJ height with an ICC-value of 0.98 (Markovic et al., 2004). The vertical ground reaction force was sampled at 1,000 Hz. For the execution of CMJ, participants were instructed to begin the jump with a downward movement, which was immediately followed by a concentric upward movement, resulting in a maximal vertical jump. During jumping, hands were kept on the hips and the depth of the downward movement was freely chosen to allow a natural movement. For the execution of DJ, participants stood in an upright position on a 37 cm box, feet shoulder-width apart, with the hands placed on the hips. Participants were asked to step off the box with their dominant leg, drop down to land evenly on both feet and jump-off the ground with a maximal-effort double-leg vertical jump. All participants were instructed to jump as high as possible (for CMJ, DJ) and to keep ground contact time as short as possible (for DJ). Two CMJ and DJ trials were completed with a rest period of 30 s between jumps. Jump height (for CMJ, DJ) as well as ground contact time and performance index (for DJ) were determined and averaged over the two trials. Jump height was calculated according to the following formula: jump height = 1/8 × g × *t*^2^, where g is the acceleration due to gravity and t is the flight time (Prieske et al., 2013). Performance index was defined as the ratio of jump height and ground contact time (Prieske et al., 2013). For each experimental condition, post-test jump performance was expressed as a percentage relative to pre-test.

### Statistical analyses

Descriptive data are presented as group mean values and standard deviations. Normal distribution was examined using the Shapiro–Wilk test. For strength and jump variables, a one-way (experimental condition: S, B+S, control) repeated measures analysis of variance (ANOVA) was used. For twitch contractile properties, a two-way (time: rested, potentiated; experimental condition: pre-test, S, B+S, control) repeated measures ANOVA was used. Homogeneity of variance was examined using the Mauchly sphericity test for repeated measures. If homogeneity was violated, the Greenhouse-Geisser correction was applied for further analyses. In *post-hoc* tests, the Bonferroni adjustment was applied to each *p-*value calculated, thereby ensuring the level of significance for all pairwise comparisons. The significance level was set at *p* < 0.05. A trend for statistical significance was deemed at *p* < 0.10. Effect sizes were calculated by converting partial eta-squared to Cohen's *d* to indicate whether a statistically significant difference is a difference of practical concern. According to Cohen (1988), the magnitude of effect sizes can be classified as small (0.2 ≤ *d* < 0.5), medium (0.5 ≤ *d* < 0.8), and large (*d* ≥ 0.8). To assess the relationship between pre-to-post-test changes in twitch contractile properties and performance measures, Pearson correlation coefficients (*r*) were calculated and classified as trivial (*r* < 0.1), small (0.1 ≤ *r* < 0.3), moderate (0.3 ≤ *r* < 0.5), large (0.5 ≤ *r* < 0.7), very large (0.7 ≤ *r* < 0.9), and almost perfect (*r* ≥ 0.9) (Hopkins et al., 2009). All analyses were performed using Statistical Package for Social Sciences (SPSS) version 24.0.

## Results

### Twitch contractile properties

**Figure 2:**
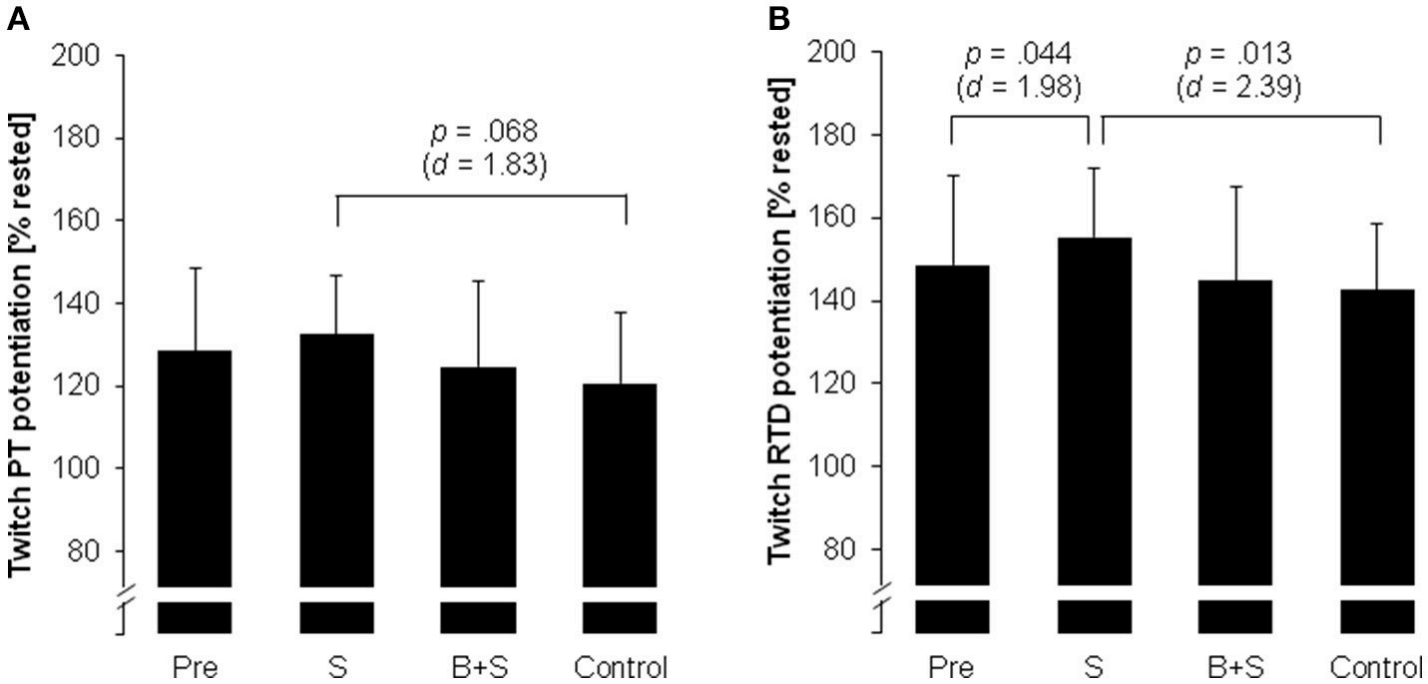
Potentiation of twitch contractile properties of the plantar flexor muscles in female elite young soccer players after the pre-test (Pre) and after the three experimental conditions [strength (S); balance and strength (B+S); passive control]. **(A)** Twitch peak torque (PT) potentiation, **(B)** twitch rate of torque development (RTD) potentiation. Values are displayed in percent relative to the pre-MVC resting twitch and in means ± standard deviation.

Significant, large-sized time by experimental condition interaction effects were observed for twitch PT (*p* < 0.05, *d* = 1.14) and twitch RTD (*p* < 0.05, *d* = 1.12). *Post-hoc* tests indicated that twitch RTD potentiation was significantly higher following S (55%) compared to pre-test (49%) and control (43%) (*p* < 0.05, 1.98 ≤ *d* ≤ 2.39; Figure 2B). A statistical trend in the direction of greater twitch PT potentiation following S (33%) compared to control (21%) was also observed (*p* = 0.068, *d* = 1.83; Figure 2A).

### Maximal voluntary strength

Table 1 shows the means and standard deviations for strength parameters. There were small-to-medium sized but non-significant effects of the experimental condition on MVC torque and voluntary RTD (*p* > 0.05, 0.33 ≤ *d* ≤ 0.64). Additionally, trivial-to-moderate correlation coefficients (−0.38 ≤ *r* ≤ 0.32) were observed between twitch contractile properties and strength variables (Table 2).

**Table 1:** Performance-related measures at pre-test (Pre) and after strength only exercise (S), balance and strength exercise (B+S), and passive control in female elite young soccer players.

**Performance measure**	**Pre**	**S**		**B+S**		**Con**		***p* value (*d*)**
		**(absolut)**	**(% Pre)**	**(absolut)**	**(%Pre)**	**(absolut)**	**(% Pre)**	
**STRENGTH PERFORMANCE**								
MVC torque (Nm)	108.3 ± 40.5	121.1 + 42.0	113.1	117.6 + 41.3	109.4	122.1 + 39.0	115.3	0.336 (0.64)
Voluntary RTD (Nm/s)	331.8 ± 187.7	394.7 + 184.3	127.7	410.1 + 185.0	135.3	379 + 146.6	129.7	0.742 (0.33)
**JUMP PERFORMANCE**								
CMJ height (cm)	22.9 ± 2.6	23.3 + 2.8	101.7	23.7 + 2.6	103.8	23.1 + 2.7	100.7	0.016 (1.36)
DJ height (cm)	17.8 ± 2.9	17.0 + 3.4	95.5	17.9 + 3.0	101.3	17.8 + 2.7	101.0	0.257 (0.72)
DJ contact time (ms)	219.1 ± 28.7	224.4 + 35.9	102.3	215.9 + 29.6	99.0	239 + 35.7	109.2	0.040 (1.17)
DJ performance index (m/s)	0.83 ± 0.20	0.78 + 0.21	94.1	0.84 + 0.16	103.6	0.77 + 0.20	93.7	0.227 (0.76)

*Absolute performance measures are presented for Pre. For S, B+S, and control, performance measures are displayed in percent relative to Pre. Values are presented in means ± standard deviation. CMJ, countermovement jump; DJ, drop jump; MVC, maximal voluntary contraction; PT, peak torque; RTD, rate of torque development*.

**Table 2:** Correlation coefficients (Pearson's *r*) between pre-to-post-test changes in twitch contractile properties and performance measures in female elite young soccer players.

**Δ Performance measure**	**Δ Twitch PT**	**Δ Twitch RTD**
**STRENGTH EXERCISE**		
MVC torque	−0.38	−0.37
Voluntary RTD	0.31	−0.10
CMJ height	0.06	−0.08
DJ height	−0.21	−0.21
DJ contact time	−0.54	−0.46
DJ performance index	0.15	0.11
**BALANCE AND STRENGTH EXERCISE**		
MVC torque	0.28	0.27
Voluntary RTD	0.32	0.18
CMJ height	0.31	0.05
DJ height	−0.15	−0.12
DJ contact time	−0.43	−0.35
DJ performance index	0.08	0.37
**CONTROL**		
MVC torque	−0.19	0.16
Voluntary RTD	−0.01	0.02
CMJ height	0.05	0.07
DJ height	−0.20	0.08
DJ contact time	−0.43	−0.61^*^
DJ performance index	0.35	0.32

* *p < 0.05*.

### Jump performance

**Figure 3:**
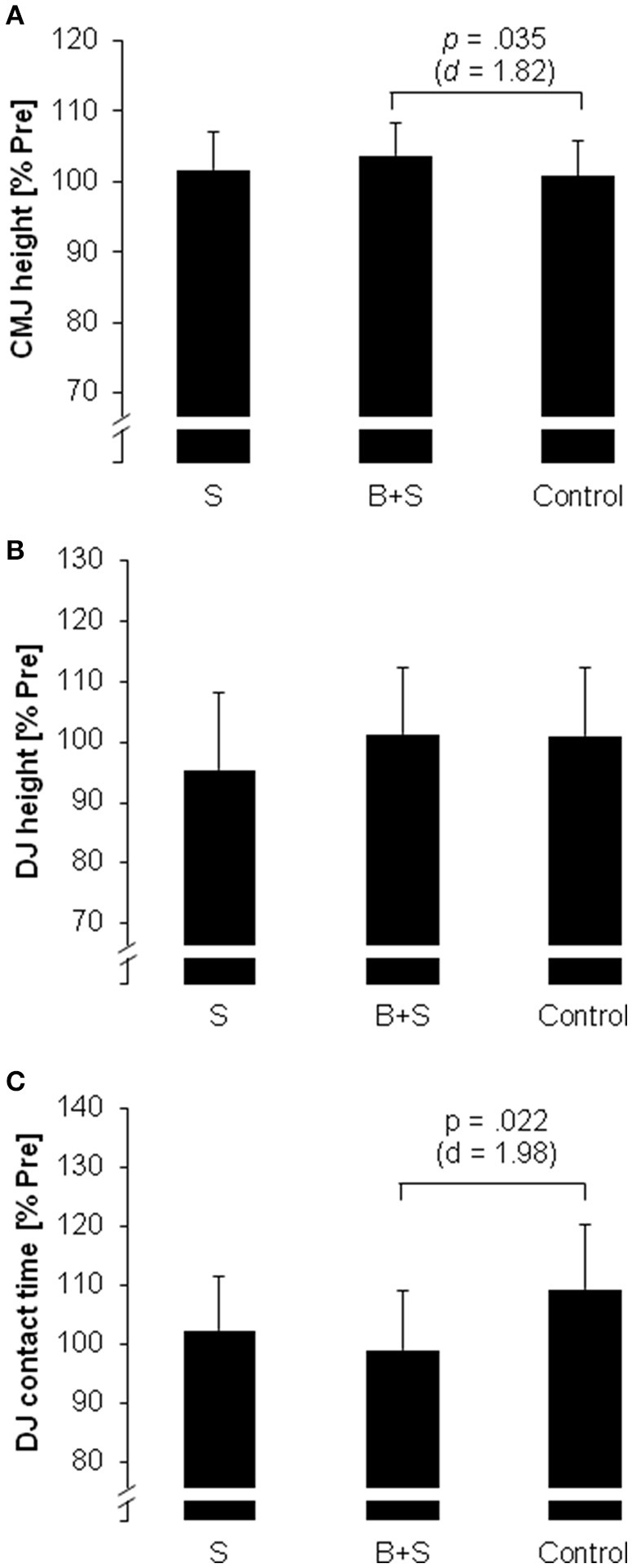
Countermovement (CMJ) and drop jump (DJ) performance in female elite young soccer players after the three experimental conditions [strength (S); balance and strength (B+S); passive control]. **(A)** CMJ height, **(B)** DJ height, **(C)** DJ ground contact time. Values are displayed in percent relative to pre-test and in means ± standard deviation.

Means and standard deviations are displayed in Table 1 for measures of jump performance. For CMJ performance, a significant and large effect of experimental condition was found for CMJ height (*p* < 0.05, *d* = 1.36). *Post-hoc* tests indicated that CMJ height was significantly larger following B+S compared to control (3%, *p* < 0.05, *d* = 1.82; Figure 3A). Additionally, a significant and large effect of experimental condition was found for DJ ground contact time (*p* < 0.05, *d* = 1.17). *Post-hoc* tests indicated that contact time was significantly lower following B+S compared to control (10%, *p* < 0.05, *d* = 1.98; Figure 3C). Further, medium-sized but non-significant effects of experimental condition were observed for DJ height and performance index (*p* > 0.05, 0.72 ≤ *d* ≤ 0.76; Figure 3B). Finally, the magnitude of correlation coefficients between twitch contractile properties and jump performance variables ranged from trivial-to-large (−0.61 ≤ *r* ≤ 0.35; Table 2).

## Discussion

To the authors' knowledge, this is the first study that investigated the acute effects of two different exercise combinations (B+S; S) vs. a passive control condition on twitch contractile properties, jump performance and maximum voluntary strength in female elite young soccer players. The main findings of this study were that (1) S exercise significantly improved plantar flexor muscle contractile properties—but not jump performance—while (2) B+S exercise significantly enhanced jump performance—but not twitch contractile properties—in female elite young soccer players.

### Effects of balance and strength exercise on PAP

It is well-documented that the contractile history of skeletal muscle directly affects its performance characteristics (Sale, 2002; Tillin and Bishop, 2009). Two antagonistic physiological processes take place during the application of a PAP protocol. First, sustained muscle contractions (whether dynamic or isometric) gradually induce muscle fatigue as indicated by decrements in performance output (Enoka and Duchateau, 2008). Second, specific muscle contractions, whether voluntary or electrically evoked, induce short-term PAP effects (i.e., improvements in twitch contractile properties). The net effect of fatigue and PAP in favor of the latter may result in enhanced strength- and power-related performance (Sale, 2002; Tillin and Bishop, 2009). Of note, findings from this study revealed significantly larger PAP (i.e., twitch RTD) following submaximal strength exercise (i.e., three sets of 8–10 leg presses at 80% 1-RM) when compared to a resting control condition (55% vs. 43%). This result is partly supported by studies that examined the effects of submaximal dynamic exercise (i.e., loaded squats) on PAP in lower limb muscles of male trained adults (Mitchell and Sale, 2011; Nibali et al., 2013; Fukutani et al., 2014). For instance, in the study of Mitchell and Sale (2011) rugby union players (mean age: 20 years) performed a set of five squats with a load corresponding to 5-RM which significantly potentiated knee extensor twitch PT (11%) compared to a passive control condition. Further, Fukutani et al. (2014) examined knee extensor twitch PT before and after submaximal squat exercises realized at moderate-intensity (45–75% 1-RM) vs. high-intensity (45–90% 1-RM) in Olympic weightlifters with a mean age of 20 years. Significant twitch PT potentiation was observed after both conditions with larger PAP following high-intensity compared to moderate-intensity exercise (40% vs. 17%). However, only few studies examined the effects of “conditioning” contractions on twitch torque potentiation in preadolescent and/or adolescent youth (Belanger and McComas, 1989; Pääsuke et al., 2000, 2003). For instance, Pääsuke et al. (2003) found significant potentiation of plantar flexor twitch peak force after 5-s MVC in boys (37%) and girls (22%) aged 9–10. Similarly, in this study, exercise-induced potentiation of plantar flexor twitch PT amounted 21–33%. Nevertheless, this is the first study to investigate the effects of submaximal dynamic strength exercise and combined balance and strength exercise on PAP in adolescent female soccer players.

Surprisingly, it has to be noted that B+S exercise did not significantly enhance plantar flexor PAP compared to S and control. We therefore hypothesize that twitch contractile properties of the plantar flexors were affected by the volume and/or type of the preceding activity (Tillin and Bishop, 2009). In fact, it was previously demonstrated that volume/duration of both maximal (Vandervoort et al., 1983; Hamada et al., 2003) and submaximal contractions (Fowles and Green, 2003; Morana and Perrey, 2009) of different lower limb muscles is an important determinant of the magnitude of PAP. For instance, Vandervoort et al. (1983) showed the largest potentiation of plantar flexor twitch PT when MVCs were sustained for 10–30 s (45%) compared to 1–3 s or 60 s (<23%) in subjects aged 21–48 years. Additionally, Morana and Perrey (2009) examined the time course of PAP during 10 min of intermittent submaximal (50% MVC) isometric knee extensions in power-trained (i.e., rugby players, weightlifters) and endurance-trained athletes (i.e., distance runners, triathletes) with a mean age of 25 years. They reported that in the rugby players/weightlifters but not in the distance runners/triathletes, twitch PT was significantly enhanced following 1 min of exercise (53%) but decreased (30% below baseline) due to fatigue after 10 min of exercise. Similarly, fatigue-related effects may have occurred following B+S exercise in our participants consisting of power-trained female young soccer players. In terms of activity type, it was argued that balance exercise can enhance neuromuscular function but not muscle contractile properties (Gollhofer, 2007). For instance, Gruber et al. (2007) examined the effects of 4 weeks of balance vs. ballistic strength training on neural (e.g., activation) and muscular adaptations (i.e., contractile properties) in healthy young adults aged 26 years. Both training programs induced specific neural adaptations (e.g., enhanced intramuscular coordination), whereas twitch contractile properties of the plantar flexor muscles remained unchanged. Thus, it can be postulated that the combination of balance and strength exercise as conducted in the present study did not provide an appropriate stimulus to enhance twitch contractile properties of the plantar flexors in female elite young soccer players. This may be attributed to neuromuscular fatigue and/or an inappropriate exercise modality of the preceding activity. It has to be acknowledged that we only examined contractile properties of plantar flexor muscles following S and B+S conditions. Previously, it was shown that PAP effects can occur in other leg muscles (e.g., knee extensors, ankle dorsal extensors) as well following submaximal and maximal lower limb exercises (Hamada et al., 2003; Morana and Perrey, 2009). Thus, future studies need to elucidate whether B+S exercise can enhance PAP effects in other lower limb muscles than plantar flexors.

### Effects of balance and strength exercise on strength and jump performance

In contrast to PAP findings, the present study revealed that jump performance (i.e., CMJ height, DJ ground contact time) was significantly improved following B+S exercise compared to the passive control condition. Additionally, MVC torque and voluntary RTD were not significantly enhanced following S or B+S exercise compared to control. Thus, it appears that the observed PAP effects induced by S exercise on the plantar flexors did not directly translate to improved strength and jump performance in female elite young soccer players. Some adult studies indicate that the potentiation of twitch contractile properties (e.g., PT) induced by submaximal and maximal contractions (Mitchell and Sale, 2011; Requena et al., 2011; Nibali et al., 2013; Fukutani et al., 2014) may partly contribute to acute performance enhancements (e.g., increased jump height). For instance, the studies of Mitchell and Sale (2011) and Fukutani et al. (2014) reported concomitant PAP-related increases in knee extensor twitch PT (11–40%) and CMJ height (3–11%) following submaximal squat exercise in trained men. The authors concluded that PAP effects contributed significantly to the gains in jump performance. However, statistical associations between pre-to-post-exercise changes of twitch contractile properties and strength/jump performance measures are inconsistent in the literature. In fact, a number of studies reported small-to-large sized correlation coefficients (−0.47 ≤ *r* < 0.50) between changes in twitch PT and CMJ height/kinetics in recreationally-trained men (Nibali et al., 2013; Pearson and Hussain, 2014) and small-to-large sized correlation coefficients (0.24 ≤ *r* ≤ 0.61) in male rugby and soccer players (Mitchell and Sale, 2011; Requena et al., 2011). Similarly, in our study correlation coefficients between exercise-induced changes in twitch PT and jump performance ranged from trivial-to-large in female elite young soccer players. Taken together, these correlation-based results indicate that individuals with greater PAP effects are not necessarily those showing the greatest strength/jump performance improvements following acute exercise.

Interestingly, the B+S exercise-induced increases in jump performance without concomitant plantar flexor PAP effects observed here may be attributed to acute neuromuscular adjustments associated to the combination of single-leg stance and leg press exercise. Acute exercise may indeed potentiate selected neuromuscular responses in lower limb muscles. More specifically, H-reflex responses of knee extensor and plantar flexor muscles were significantly potentiated 3–10 min after maximal contractions in adults (Guellich and Schmidtbleicher, 1996; Trimble and Harp, 1998; Folland et al., 2008). Such potentiation at the spinal level appears to be due to the recruitment of larger motor units (Tillin and Bishop, 2009). Additionally, there is evidence that balance exercise has the potential to enhance neuromuscular function (e.g., afferent feedback, motor unit recruitment) during lower limb activities (Gruber et al., 2007; Horslen et al., 2013). For instance, Horslen et al. (2013) reported larger muscle spindle sensitivity under unstable compared to stable conditions. The authors stated that these adaptations may increase afferent feedback to cortical and/or subcortical areas during postural challenges. Further, Gruber et al. (2007) found significant increases in voluntary RTD of the plantar flexors and EMG median frequency of the triceps surae muscle following 4 weeks of balance training in healthy young adults. These authors argued that the observed training-induced RTD improvements are most likely caused by increases in EMG median frequency which is indicative of improved intramuscular coordination (e.g., earlier recruitment of larger motor units). Notably, it was speculated that enhanced recruitment of higher order motor units as a result of preceding muscle activity might increase the contribution of fast fibers to muscle contraction and finally to performance (Tillin and Bishop, 2009). Thus, it is plausible to postulate that short-term neuromuscular adjustments associated to B+S exercise are responsible, at least in part, for the observed enhancements in jump performance.

## Conclusions

Findings from the present study revealed short-term improvements in twitch contractile properties following S but not B+S exercise compared to a passive control condition. In contrast, significant increases in jump performance were observed following B+S but not S exercise with respect to control. Thus, it can be concluded that submaximal leg press strength exercise successfully induced PAP effects in the plantar flexor muscles of female elite young soccer players. However, plantar flexor PAP appears to be affected by the type and/or volume of the preceding activity (i.e., S vs. B+S exercise). Further, PAP effects in the plantar flexor muscles did not directly translate into jump performance improvements, probably because these latter rely more on neuromuscular adjustments (e.g., intramuscular coordination) rather than on intrinsic muscle properties. From a practical point of view, the sequencing of balance and strength exercises (e.g., leg press, loaded squats) is recommended as a procedure to acutely enhance jump performance (e.g., during complex training) in female young athletes.

## Author contributions

OP, NM, and UG made substantial contributions to conception, design, and data collection; OP contributed to data acquisition and carried out data analysis and interpretation together with NM and UG; OP wrote the first draft of the manuscript and all authors were involved in revising it critically for important intellectual content; OP, NM, and UG gave final approval of the version to be published and agreed to be accountable for all aspects of the work.

### Conflict of interest statement

The authors declare that the research was conducted in the absence of any commercial or financial relationships that could be construed as a potential conflict of interest.
